# Inhibition of the NLRP3 Inflammasome by a Quercus Serrata Extract and Isolation of the Component Compounds for the Treatment of Arthritis

**DOI:** 10.1155/2022/4428269

**Published:** 2022-12-28

**Authors:** Seo Yeon Seonu, Min Ji Kim, Min Won Lee

**Affiliations:** Laboratory of Pharmacognosy and Natural Product Derived Medicine, College of Pharmacy, Chung-Ang University, Seoul 06974, Republic of Korea

## Abstract

*Quercus serrata* belongs to the Fagaceae family. There are 600 known species of *Quercus* worldwide. *Q. serrata* is distributed nationally in Korea, Japan, and China and grows to a height of 10–15 m. It exhibits a light grey bark with longitudinal furrows; the leaves are 6–12 cm long and 2.5–5 cm wide. The *Quercus* genus reportedly exhibits several types of bioactivity, including antioxidant, anti-inflammatory, antifungal, antimicrobial, and anticancer activity. Additionally, it has been reported that *Quercus* produces diverse phytochemicals, including tannins, flavonoids, and triterpenoids. Herein, we describe the column chromatographic isolation of five compounds from a *Q. serrata* extract. The compounds included caffeic acid (1), myricetin-3-O-cellobioside (2), phloroglucinol (3), (S)-2,3-HHDP-D-glucopyranoside (4), and pedunculagin (**5**). We assessed the 2,2-diphenyl-1-picryl-hydrazyl (DPPH) radical scavenging activity, antioxidant activity, NLR family pyrin domain-containing 3 (NLRP3) inflammasome (including NLRP3, ASC, and caspase-1) inhibitory effects, and collagenase inhibition activity of the *Q. serrata* extract and its constituent compounds. Our results indicated that the *Q. serrata* extract and the isolated constituent compounds showed inhibitory activity with reference to nitric oxide production, inflammasome component expression, and collagenase activity. Our findings imply that the *Q. serrata* extract and the isolated constituent compounds are potential candidates for the treatment of inflammatory diseases such as arthritis.

## 1. Introduction

The *Quercus* genus is part of the Fagaceae family, with 600 known species worldwide. Several variants of this species exist in Korea, including *Q. acutissima*, *Q. variabilis*, *Q. dentata*, *Q. aliena*, *Q. mongolica*, *Q. serrata*, *Q. acuta*, *Q. glauca*, *Q. myrsinifolia*, *Q. salicina*, and *Q. gilva*. The species *Q. serrata* is widely distributed throughout Korea, Japan, and China and can reach up to 10–15 m in height. The bark is light grey in color with longitudinal furrows. The leaves are 6–12 cm long and 2.5–5 cm wide, with light grey elongated oval patterns on the underside [1]. The *Quercus* genus reportedly exhibits several types of bioactivity, including antioxidant [2, 3], anti-inflammatory [4–7], antifungal [8–10], antidiabetic [11, 12], antimicrobial [13, 14], anticancer [15], anti-hepatoprotective [16], antibacterial [17], and anti-urolithiatic [18] activities. Phytochemical studies of *Quercus* spp. revealed that it produces flavonoids [4, 5, 19], triterpenoids [20, 21], and tannins [22–24]. Natural products have historically contributed to the treatment of various diseases. Scientific developments in recent years have demonstrated the applicability of natural products in various fields, and this has led to increased scientific interest in such products [25–29]. Several natural products have been shown to exert inflammasome inhibitory activity [30, 31]. Consequently, there has been an increasing amount of research into the use of natural products as pharmacotherapeutics and nutraceuticals for cancer, gastrointestinal disorders, nociceptive pain, and anxiety disorders; they have also been explored for application such as aphrodisiacs [32–38]. In this respect, the inflammasome inhibitory profile of natural products is an interesting property that can be applied to develop phytochemical treatments for arthritis. However, studies investigating the application of natural products for arthritis treatment via the modulation of the NLR family pyrin domain-containing 3 (NLRP3) inflammasome have not yielded convincing evidence, and studies on *Q. serrata* in this context are especially rare. We previously investigated the use of a *Quercus* spp. Extract as a therapeutic agent for inflammatory diseases by evaluating nitric oxide (NO) production and proinflammatory cytokine levels [18] determined that such extracts can be applied for treating inflammatory diseases such as arthritis. Inflammasomes are cytosolic multi-protein complexes comprising a nucleotide-binding domain and leucine-rich repeat-containing proteins (NLRs), caspase-1, and the adapter protein apoptosis-associated speck-like protein containing a caspase recruitment domain (ASC). Inflammasomes activate inflammatory responses and may trigger the maturation of proinflammatory cytokines such as interleukin (IL)-18 and IL-1*β* [39]. The NLRP3 inflammasome contains the NLRP3 sensor, an ASC adaptor, and caspase-1 protease. Following its assembly into large cytoplasmic complexes and caspase-1 activation, NLRP3 catalyzes the maturation and secretion of IL-18 and IL-1*β*. In addition to cytokine production, NLRP3 inflammasome activation may also facilitate pyroptosis (caspase-1-mediated rapid cell death) [40, 41]. Inflammasomes can cause inflammatory disorders such as arthritis, multiple sclerosis, Alzheimer's disease, atherosclerosis, type 2 diabetes, and systemic lupus erythematosus [42]. Arthritis is a chronic inflammatory disease that is caused by diverse triggers. Numerous studies have sought to identify the correlation between arthritis and inflammasomes. The levels of NLRP3, ASC, and caspase-1 increased in both arthritic rats and patients with arthritis [43]. In this study, we demonstrate that a *Q. serrata* extract and its constituent compounds can downregulate the expression of the NLRP3 inflammasome proteins and show activity as antioxidants, anti-inflammatory agents, and collagenase inhibitors. Based on this bioactivity profile, we propose that this extract and its constituent compounds can potentially be applied for the treatment of inflammatory diseases such as arthritis.

## 2. Materials and Methods

### 2.1. Plant Materials

*Q. serrata* leaves were collected from Pocheon, Republic of Korea, in May 2020. The species was identified by Dr. Sung Sik Kim. A voucher specimen was stored at the Herbarium of the College of Pharmacy, Chung-Ang University.

### 2.2. General Experimental Procedure

Column chromatography was conducted using MCI gel CHP 20P (75–150 *μ*m, Mitsubishi Chemical, Tokyo, Japan), ODS-B gel (40–60 *μ*m, Daiso, Osaka, Japan), and Sephadex LH-20 (10–25 *μ*m, GE Healthcare Bio-Science AB, Uppsala, Sweden).

A pre-coated silica gel 60 F254 plate (Merck, Darmstadt, Germany) was used to perform thin-layer chromatography (TLC) with a mixture of chloroform, methanol, and distilled water (6 : 4:1, volume ratio) and a mixture of benzene, ethyl formate, and formic acid (1 : 7:2, volume ratio). A 10% solution of sulfuric acid (H_2_SO_4_) or anisaldehyde-H_2_SO_4_ was heated and sprayed with ferric chloride (FeCl_3_) to determine the location of the stains on the chromatograph.

To clarify the structure of the isolated compounds, 1D-nuclear magnetic resonance (NMR) analyses such as 1H-(600 MHz) and ^13^C-(150 MHz) NMR were performed and recorded using JEOL (JEOL, Massachusetts, USA) at Chung-Ang University.

### 2.3. Extraction and Isolation

Extracts were obtained from 2.2 kg of *Q. serrata* leaves using 80% acetone at room temperature. Such extracts were obtained three times over the course of three days. The extracts were concentrated by removing the acetone under vacuum, and this procedure yielded 350.12 g of concentrated extract. The *Q. serrata* extract (300 g) was then filtered using Celite 545, and the remaining extract was stored at -20°C. The filtered *Q. serrata* extract was loaded onto a Sephadex LH-20 chromatography column with a methanol: water (MeOH : H_2_O) gradient solvent system (ratio between 0 : 10 and 10 : 0) and yielded 12 subfractions (*Q. serrata*-1–12).

The fractionation of *Q. serrata*-8 (44.97 g) during repeated column chromatography using the MCI gel CHP 20P with a gradient solvent system of MeOH : H_2_O (from 0 : 10 to 10 : 0) yielded 24 subfractions. The *Q. serrata*-8-12 subfractions (494.9 mg) were loaded onto octadecyl silica (ODS) gel with a gradient solvent system of MeOH : H_2_O (from 0 : 10 to 10 : 0) and yielded compounds 1 (caffeic acid, 34.5 mg) and 2 (myricetin-3-O-cellobioside, 52.8 mg).

The fractionation of *Q. serrata*-7 (7.7 g) during repeated column chromatography using the MCI gel with a gradient solvent system of MeOH : H_2_O (from 0 : 10 to 10 : 0) yielded compound **3** (phloroglucinol, 94.8 mg).

*Q. serrata*-9 (17.01 g) was loaded onto an MCI gel CHP 20P column with a gradient solvent system including MeOH : H_2_O (from 0 : 10 to 10 : 0) and produced 22 subfractions. *Q. serrata*-9-5 yielded four subfractions when loaded onto an ODS column with a gradient solvent system including MeOH : H_2_O (from 0 : 10 to 10 : 0). Column chromatography was repeated for *Q. serrata*-9-5-1 (1.55 g) using Sephadex LH-20 with a gradient solvent system including MeOH : H_2_O (from 0 : 10 to 10 : 0), yielding compounds 4 ((S)-2,3-HHDP-D-glucopyranoside, 21.3 mg) and 5 (pedunculagin, 897.1 mg).

### 2.4. High-Performance Liquid Chromatography (HPLC) Analysis

The constituents of the *Q. serrata* extract were analyzed by HPLC. The mobile phase consisted of solvent A (0.2% acetic acid in H_2_O) and solvent B (acetonitrile, ACN) (Table 1). The *Q. serrata* extract and the isolated constituent compounds were dissolved in 100% MeOH.

### 2.5. DPPH Radical Scavenging Assay

A DPPH radical scavenging assay was used to evaluate antioxidant activity. A 20 *μ*L aliquot of each sample was dissolved in anhydrous ethanol and added to 180 *μ*L of 0.2 mM DPPH (Sigma-Aldrich, St. Louis, MO, USA) solution. The mixture was incubated for 37°C, 30 min, and the absorbance was measured at 517 nm using an enzyme-linked immunosorbent assay reader (Tecan Co., Ltd., Salzburg, Austria). The DPPH free radical scavenging activity was calculated in terms of percent inhibition (%). Half-maximal inhibitory concentration (IC_50_) values were defined as the concentrations that could scavenge 50% of the DPPH free radicals. L-ascorbic acid was used as the positive control. Inhibitory activity was calculated as follows:

(1) $$\begin{array}{c} \text{Percent inhibition}\left(\%\right)=100=\left(\frac{\text{sample }O.D.}{\text{control }O.D.}\right)\times 100\;O.D\text{ Optical Density.} \end{array}$$

### 2.6. Cell Culture

RAW 264.7 cells were purchased from the Korean Cell Line Bank. The cells were grown at 37°C in a humidified atmosphere (with approximately 5% CO_2_) in Dulbecco's modified Eagle's medium (DMEM) (Corning, NY, USA) supplemented with 10% fetal bovine serum (Welgene, Gyeongsangbuk-do, Korea), 100 IU/mL penicillin G, and 100 mg/mL streptomycin (Gibco BRL, Grand Island, NY, USA).

### 2.7. Cell Viability Assay

The cytotoxicity of the test extract/compounds was determined prior to the biological assay based on the mitochondria-dependent reduction of 3-(4,5-dimethylthiazol-2-yl)-2,5-diphenyltetrazolium-bromide (MTT) (Sigma-Aldrich) to formazan. RAW 264.7 cells (2 × 105 cells/well) were seeded in a 96-well plate and incubated at 37°C for 16 h, treated with 20 *μ*L of serum-free DMEM, and incubated for 24 h at 37°C. The medium was then removed, and 100 *μ*L of MTT solution (0.5 mg/mL) was added to each well. After 4 h of incubation, the supernatant was aspirated. Finally, 100 *μ*L of dimethyl sulfoxide (DMSO) was added to each well to dissolve the formazan crystals, and the absorbance at 540 nm was measured using an ELISA reader (Tecan Co., Ltd.). Relative cell viability was evaluated based on the quantity of MTT converted to the insoluble formazan salt. Distilled water served as the control sample. The optical density of the formazan generated in the control cells was considered to represent 100% viability. The data are expressed as the mean percentage of viable cells versus the control.

(2) $$\begin{array}{c} \text{Cell viability }\left(\%\right)=\frac{\text{sample }O.D.}{\text{control }O.D.}\times 100. \end{array}$$

### 2.8. NO Production Assay

RAW 264.7 murine macrophage cells (2 × 105 cells/well) were seeded in 96-well plates and pre-incubated for 16 h at 37°C in a humidified atmosphere (with approximately 5% CO_2_). The cells were then incubated in serum-free medium containing the test compound/extract and 1 *μ*g/mL of lipopolysaccharide (LPS) (Sigma-Aldrich). After incubating for an additional 24 h, the NO content was evaluated using a Griess assay. For the Griess assay, 100 *μ*L of Griess reagent (Sigma-Aldrich) was added to 100 *μ*L of supernatant from the treated cells. The absorbance of the samples was recorded at 540 nm [44]. NG-monomethyl-L-arginine monoacetate salt (L-NMMA) was used as a positive control. The IC50 value was defined as the concentration that could inhibit 50% of NO production. The inhibitory activity with reference to NO production was calculated as follows:

(3) $$\begin{array}{c} \text{Inhibition }rate\left(\%\right)=100-\frac{\left(\text{sample }O.D.-\text{blank }O.D.\right)}{\left(\text{control }O.D.-\text{blank }O.D.\right)}\times 100. \end{array}$$

### 2.9. Western Blot Analysis

RAW 264.7 macrophage cells (1 × 106 cells/well) were pre-incubated for 16 h at 37°C and treated with 1 *μ*g/mL of LPS for 24 h. The treated cells were harvested and washed twice with phosphate-buffered saline. Cell lysates were then prepared using radio-immunoprecipitation assay buffer (150 mM NaCl, 50 mM Tris-HCl (pH 7.4), 0.1% sodium dodecyl sulfate (SDS), 1% Triton X-100, and 1 mM ethylenediaminetetraacetic acid) (Thermo Fisher Scientific, MA, USA). The cell lysates were centrifuged at 13,000 rpm for 15 min at 4°C, and the supernatants (protein: 60 *μ*g) were electrophoresed using SDS-polyacrylamide gels (10% and 12%). The separated proteins were then transferred to a polyvinylidene fluoride (PVDF) membrane (Bio-Rad, CA, USA). The membrane was blocked using a blocking buffer (Thermo Fisher Scientific) for 30 min at room temperature. Then, the membrane was incubated overnight at 4°C with antibodies NLRP3 (1 : 1000; Cell Signaling, MA, USA), ASC (1 : 100; Santa Cruz, CA, USA), and caspase-1 (1 : 1000; Cell Signaling). After washing the membranes with Tris-buffered saline-Tween (TBS-T) (Bio-Rad, CA, USA) three times, they were incubated with horseradish peroxidase (HRP)-linked anti-rabbit IgG secondary antibody (1 : 3000; Cell Signaling) and m-IgG*κ* binding protein (BP)-HRP (1 : 1000; Santa Cruz) for 1 h at room temperature. The bands were visualized using a LAS-500 luminescent image analyzer (GE Healthcare Life Sciences, NJ, USA) after treating the membrane with ECL detection reagent (GE Healthcare Life Sciences).

### 2.10. Collagenase Assay

Collagenase assay was conducted using a previously described procedure [45]. Collagenase (5 *μ*g) and 4-phenylazobenzyloxycarbonyl-L-Pro-L-Les-Gly-L-Pro-D-Arg (PZ-peptide, 0.5 mg) (a substrate of collagenase) were incubated with or without samples in 0.1 M Tris buffer (pH 7.4) at 37°C for 30 min (total volume 1.7 mL). To terminate the enzyme reaction, 1 mL of 25 mM citric acid solution was added. After mixing with 5 mL of ethyl acetate, the absorbance of the organic layer was measured at 320 nm. Epigallocatechin gallate (EGCG) was used as the positive control. The inhibitory activity was calculated as follows:

(4) $$\begin{array}{c} \text{Inhibitory activity}\left(\%\right)=\left[\frac{\left(O.D.\text{control}-O.D.\text{sample}\right)}{O.D.\text{control}}\right]\times 100. \end{array}$$

^*∗*^O.D. control: O.D. of control with collagenase - O.D. of control without collagenase ^*∗*^O.D. sample: O.D. in the presence of test sample with collagenase - O.D. in the presence of test sample without collagenase.

### 2.11. Statistical Analysis

All data are expressed as mean ± SD. One-way analysis of variance (ANOVA) and the Student–Newman–Keuls test were performed using the Statistical Package for Social Sciences (SPSS 24) software. The resulting values were considered significantly different at *p* < 0.05.

## 3. Results

### 3.1. Isolation and Structural Identification of the Compounds

**Figure 1:**
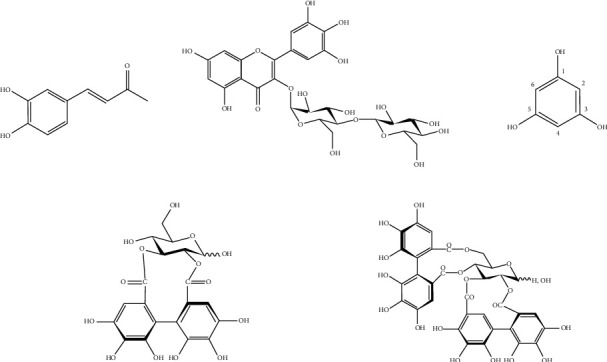
Structures of compounds 1–5.

Chromatographic analysis of the *Q. serrata* extract using Sephadex LH-20, MCI gel CHP–20P, and ODS-B gel yielded five compounds (1–5). These compounds were identified as caffeic acid (1), myricetin-3-O-cellobioside (2), phloroglucinol (3), (S)-2,3-HHDP-D-glucopyranoside (4), and pedunculagin (5) based on instrumental analysis and comparisons with references (Figure 1). This is the first time these compounds (1–5) have been isolated from *Q. serrata*. ^1^H and ^13^C-NMR spectra of the compounds (1–5) are included in Supplementary Material as Figures S1–S5.

#### 3.1.1. Compound **1**

**1** was a brown powder. A purple spot was detected by spraying the TLC plate with 10% H_2_SO_4_ followed by heating, and a black spot was detected using FeCl_3_.

The 1H-NMR spectrum of **1** revealed ABX-type protons (*δ* 7.12 (1H, *d*, *J* = 1.8 Hz, H-2), 6.95 (1H, dd, *J* = 7.8, 1.8 Hz, H-6), 6.82 (1H, *d*, *J* = 7.8 Hz, H-5)) and two doublet signals with high *J* values, indicating trans-type protons (*δ* 7.48 (1H, *d*, *J* = 16.2 Hz, H-7), 6.21 (1H, *d*, *J* = 16.2 Hz, H-8)).

By comparing the spectral data with those from previous literature [46], the structure of 1 was identified as caffeic acid.

#### 3.1.2. Compound 2

**2** was a brown powder. A black spot was detected using FeCl_3_, and a yellow spot was detected by spraying the TLC plate with 10% H_2_SO_4_ followed by heating.

The 1H-NMR spectrum of **2** revealed a myricetin moiety (*δ* 7.15 (2H, *s*, H-2′, 6′), 6.34 (1H, *m*, H-8), 6.16 (1H, *m*, H-6)) and two anomeric proton signals (*δ*5.43 (1H, *d*, *J* = 7.8 Hz, H-1″) and 5.31 (1H, *d*, *J* = 7.2 Hz, H-1‴)).

The 13C-NMR spectrum of **2** also showed myricetin moiety (*δ* 177.92 (C-4), 164.62 (C-7), 161.76 (C-5), 156.78 (C-9), 156.70 (C-2), 145.89 (C-3′, 5′), 137.16 (C-4′), 134.00 (C-3), 120.56 (C-1′), 109.05 (C-2′, 6′), 104.48 (C-10), 99.15 (C-6), and 93.88 (C-8)). The presence of a 3-O linkage was indicated by a 9.89 ppm downshift of C-2 (*δ* 156.70) and a 2.18 ppm upshift of C-3 (*δ* 134.00). A cellobiose moiety (*δ* 102.52 (C-1‴), 101.38 (C-1″), 78.13 (C-4″), 77.08 (C-5‴), 76.44 (C-3‴), 74.44 (C-2‴), 73.79 (C-3″), 71.72 (C-2″), 70.42 (C-5″), 68.52 (C-4‴), 61.59 (C-6‴), and 60.56 (C-6″)) was observed with a 9 ppm downshift of C-1”; this indicated that C-1″ has a linkage with the 3-hydroxyl group of myricetin.

By comparing the results with data from previous literature [47, 48], the structure of 2 was identified as myricetin-3-O-cellobioside.

#### 3.1.3. Compound 3

**3** was a brown powder. A grey spot was detected by spraying the TLC plate with 10% H_2_SO_4_ followed by heating, and a dark blue spot was detected using FeCl_3_.

The 1H-NMR spectrum of 3 showed only one signal at *δ* 6.87, and the 13C-NMR spectrum showed two signals (*δ* 145.90 (C-1, 3, 5) and 109.27 (C-2, 4, 6)).

By comparing our results with those from previous literature [49], the structure of 3 was identified as phloroglucinol.

#### 3.1.4. Compound 4

4 was a brown powder. A dark pink spot was detected by spraying the TLC plate with 10% H_2_SO_4_ followed by heating, and a dark blue spot was detected using FeCl_3_.

The 1H-NMR of 4 showed duplicated signals below 5.34 ppm; thus, it is possible that **4** may have two isomers on an anomeric center on the sugar. In addition, the 1H-NMR spectrum of **4** revealed one hexahydroxydiphenoyl (HHDP) signal (*δ* 6.65, 6.64, 6.56, 6.55 (4H in total, *s*, HHDP-H)) in the aromatic region and sugar protons existing as *α* and *β* forms in the sugar region. The 13C-NMR spectrum of 4 revealed four signals in the aromatic region indicating–COO (*δ* 169.97, 169.94, 169.40, 169.20) and included HHDP and two isomer sugar forms in the sugar region (*δ* 93.92, 90.58, 79.89, 77.58, 77.17, 77.07, 74.82, 71.96, 67.43, 67.21, 61.00, 60.85).

By comparing these results to NMR data from previous literature [50], the structure of 4 was identified as (S)-2,3-HHDP-D-glucopyranoside.

#### 3.1.5. Compound 5

**5** was a brown amorphous powder. A brown spot was detected by spraying the TLC plate with 10% H_2_SO_4_ followed by heating, and a dark blue spot was detected using FeCl_3_.

Anomeric proton signals below 5.45 ppm were observed on the 1H-NMR spectrum of 5, and all signals were duplicated. Thus, 5 is thought to be a mixture of two isomers caused by unacylated anomeric centers on a sugar. The 1H-NMR spectrum also revealed two HHDP moieties (*δ* 6.64, 6.63, 6.57, 6.57, 6.53, 6.48, 6.30, 6.29 (8H in total, each *s*, HHDP-H)) in the aromatic region and a glucose core with 4C1 conformation (*δ* 3.74–5.45 (large coupling constants)) in the sugar region.

By comparing these results with data from previous literature [51], the structure of 5 was identified as pedunculagin.

### 3.2. HPLC Analysis

**Figure 2:**
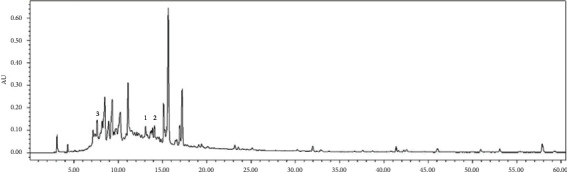
HPLC chromatogram of the *Q. serrata* extract obtained at 254 nm.

**Figure 3:**
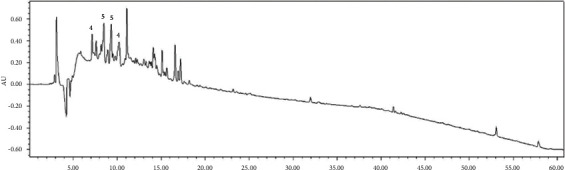
HPLC chromatogram of the *Q. serrata* extract obtained at 220 nm.

Contents analysis of the *Q. serrata* extract was conducted using HPLC. The composition of the extract was found to be as follows: compound 1 0.04%, compound 2 0.32%, compound 3 0.22%, compound 4 6.22%, and compound 5 16.56%. The results showed that the major component was compound **b,** which comprised 16.56% of the QS extract (Figures 2 and 3, Table 2).

The antioxidant ability of the *Q. serrata* extract and its component compounds (1–5) was evaluated using the DPPH radical scavenging activity. The DPPH free radical is deep purple in color and absorbs light at 517 nm. It loses electrons upon reacting with antioxidants, resulting in a reduced form, which is yellow in color. The reaction is thus monitored by measuring the absorbance at 517 nm.

**Figure 4:**
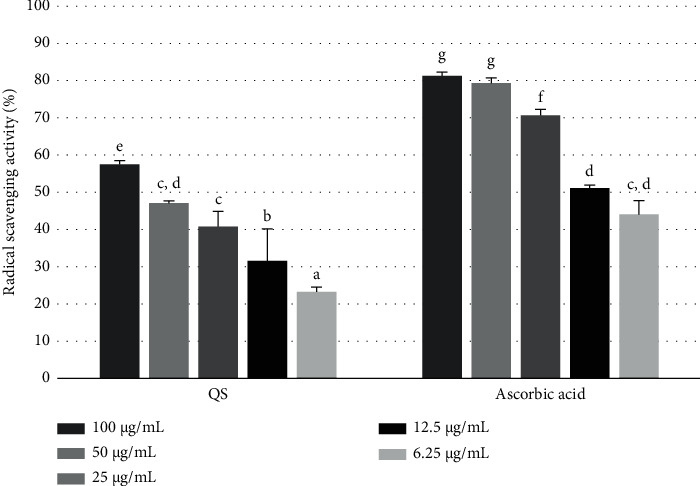
2,2-diphenyl-1-picryl-hydrazyl (DPPH) radical scavenging activity of the *Q. serrata* extract and ascorbic acid at concentrations of 100, 50, 25, 12.5, and 6.25 *μ*g/mL. Values are expressed as the mean ± SD of triplicate experiments. Values in a particular column indicated with different superscripts (a–g) are statistically different (*p* < 0.05).

**Figure 5:**
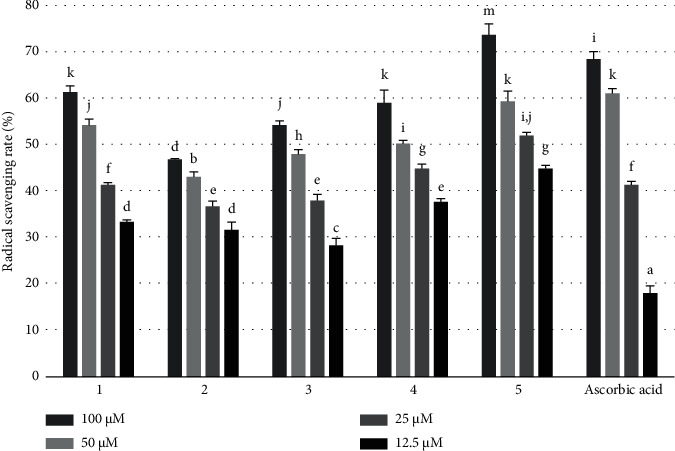
2,2-diphenyl-1-picryl-hydrazyl (DPPH) radical scavenging assay of compounds 1–5 and ascorbic acid at concentrations of 100, 50, 25, 12.5, and 6.25 *μ*g/mL. Values are expressed as the mean ± SD of triplicate experiments. Values in a particular column indicated with different superscripts (a–g) are statistically different (*p* < 0.05).

The IC_50_ of the test compounds was calculated by plotting the inhibitory activity (%) in the DPPH free radical scavenging assay against the concentration. A low IC_50_ value indicates a high antioxidant activity. The *Q. serrata* extract (IC_50_ = 65.84 ± 5.79 *μ*g/mL) demonstrated superior radical scavenging activity compared with ascorbic acid (IC_50_ = 11.20 ± 5.97 *μ*g/mL) (Figure 4, Table 3). Compounds 1, 4, and 5 also exhibited potent radical scavenging activity compared with ascorbic acid (IC_50_ = 51.07 ± 1.47 *μ*M). In particular, compound **5** (IC_50_ = 24.18 ± 1.06 *μ*M) exhibited the strongest activity compared with ascorbic acid (Figure 5, Table 4).

### 3.3. MTT Cell Viability Assay

The effect of the *Q. serrata* extract on cell viability and its cytotoxic activity were evaluated by the mitochondria-dependent reduction in MTT. When MTT is processed in living cells, it is reduced by the reductase in the mitochondria, forming formazan crystals. The formation of formazan indicates a lack of cytotoxicity.

**Figure 6:**
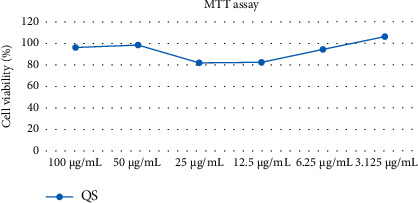
Effects of the *Q. serrata* extract (QS) at concentrations of 100, 50, 25, 12.5, 6.25, and 3.125 *μ*g/mL on the viability of RAW 264.7 cells.

The MTT assay was performed with the *Q. serrata* extract at concentrations of 100, 50, 25, 12.5, 6.25, and 3.125 *μ*g/mL in RAW 264.7 cells. The cell viability was maintained at >80% at all concentrations of the extract. These results demonstrate that the inhibition of NO production and the production of inflammatory molecular products due to treatment with *Q. serrata* do not cause cytotoxicity (Figure 6).

### 3.4. Inhibition of NO Production

The anti-inflammatory activity of the *Q. serrata* extract and the isolated component compounds 1–5 was evaluated based on the inhibition of NO production in RAW 246.7 macrophage cells.

NO is biosynthesized from L-arginine by three nitric oxide synthase (NOS) enzyme isoforms (nNOS, eNOS, and iNOS). NO regulates the cellular and toxic responses. However, excessive NO production is considered to enhance tumor development and DNA methylation. Thus, the inhibition of inflammatory response-related NO production might be a useful therapeutic and prophylactic method in arthritis.

**Figure 7:**
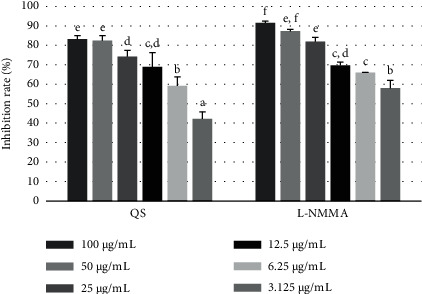
Inhibitory effect of the *Q. serrata* extract and positive control L-NMMA at concentrations of 100, 50, 25, 12.5, and 6.25 *μ*g/mL on NO production in LPS-stimulated RAW 264.7 cells. Values are expressed as the mean ± SD of triplicate experiments. Values with different superscripts (a–g) in the same columns are statistically different (*p* < 0.05). L-NMMA, NG-monomethyl-L-arginine monoacetate; QS, *Q.* serrata extract; LPS, lipopolysaccharide; NO, nitric oxide; SD, standard deviation.

**Figure 8:**
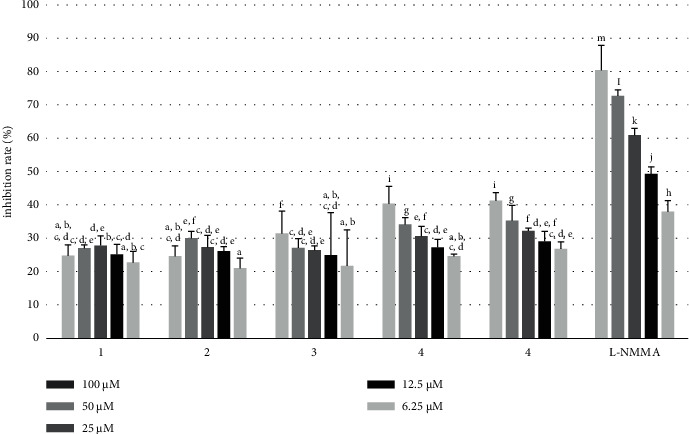
Inhibitory effect of compounds 1–5 on nitric oxide (NO) production in lipopolysaccharide (LPS)-stimulated RAW 264.7 cells. L-NMMA, NG-monomethyl-L-arginine monoacetate.

The *Q. serrata* extract showed a potent inhibitory effect on NO production (IC_50_ = 6.32 ± 0.19 *μ*g/mL); the activity was comparable to that of the positive control L-NMMA (IC_50_ = 5.10 ± 0.14 *μ*g/mL) (Figure 7, Table 5). Compound **4** (117.66 ± 2.63 *μ*M) and compound **5** (111.79 ± 2.52 *μ*M) showed good inhibition of NO production compared with that of the other compounds (Figure 8, Table 6).

### 3.5. Inhibitory Effects on Inflammasome Protein Expression

The inhibitory effects of the *Q. serrata* extract and ellagitannin (pedunculagin, compound **5**) on the expression of the components of the NLRP3 inflammasome (NLRP3, ASC, and caspase-1) in RAW 264.7 cells were evaluated using Western blot analysis. The NLRP3 complex is considered important as a treatment target for inflammatory diseases because it controls inflammatory cytokine levels.

**Figure 9:**
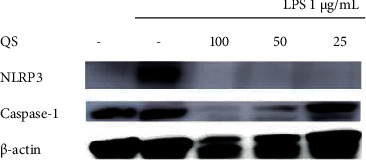
Effects of the *Q. serrata* extract (QS) on the expression of NLRP3 and caspase-1 in LPS-stimulated RAW 264.7 cells. 100, 100 *μ*g/mL; 50, 50 *μ*g/mL; 25, 25 *μ*g/mL; LPS, lipopolysaccharide; NLRP3, NLR family pyrin domain-containing 3.

**Figure 10:**
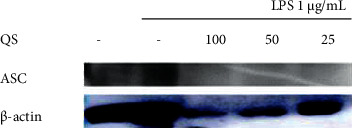
Effects of the *Q. serrata* extract (QS) on the expression of ASC in LPS-stimulated RAW 264.7 cells. 100, 100 *μ*g/mL; 50, 50 *μ*g/mL; 25, 25 *μ*g/mL; ASC, adapter protein apoptosis-associated speck-like protein containing a caspase recruitment domain; LPS, lipopolysaccharide.

**Figure 11:**
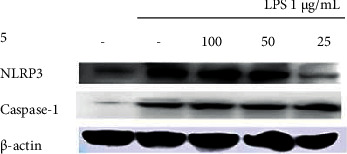
Effects of compound 5 on the expression of NLRP3 and caspase-1 in LPS-stimulated RAW 264.7 cells. 100, 100 *μ*M; 50, 50 *μ*M; 25, 25 *μ*M; LPS, lipopolysaccharide; NLRP3, NLR family pyrin domain-containing 3.

**Figure 12:**
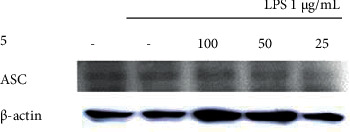
Effects of compound 5 on the expression of ASC in LPS-stimulated RAW 264.7 cells. 100, 100 *μ*M; 50, 50 *μ*M; 25, 25 *μ*M; LPS, lipopolysaccharide; ASC, adapter protein apoptosis-associated speck-like protein containing a caspase recruitment domain.

The expression of NLRP3 in LPS-treated cells incubated with the *Q. serrata* extract was lower than that in the control group, indicating that the extract inhibited NLRP3 protein expression. Similarly, compound 5 also inhibited the expression of NLRP3, caspase-1, and ASC (level compared with that in the control group) (Figures 9–12).

### 3.6. Inhibition of collagenase

The inhibitory effects of the *Q. serrata* extract and ellagitannin (pedunculagin, compound **5**) on collagenase activity were evaluated. The cartilage tissue associated with arthritis includes collagen and various proteins. Collagen plays an important role in joints, and collagenase is the key enzyme that cleaves and breaks down collagen.

Our results indicated that the *Q. serrata* extract (99.68%) and compound 5 (57.52%) showed potent inhibitory activity against collagenase compared with the positive control, EGCG (85.30%), at 25 *μ*M (Tables 7, 8 and 9).

## 4. Discussion

We aim to evaluate an extract made from *Q. serrata* with reference to inflammasome inhibition, antioxidant, anti-inflammatory, and collagenase inhibition activities. Inflammasomes are multi-protein complexes that regulate the secretion of inflammatory cytokines such as IL-1 and IL-18. They are known to be key mediators of inflammation and immunity. When inflammatory cytokines are overexpressed due to inflammasome regulatory disorders, pyroptosis (a type of apoptosis) is induced, and various chronic inflammatory diseases such as diabetes, inflammatory bowel disease, arthritis, and prostate hypertrophy (please add a space between the concentration numeral and the “uM” in the X-axis labels) occur [39–42]. Recently, various types of inflammasomes have been studied, of which the NLRP3 complex is considered very important as a treatment target for inflammatory diseases. This is because this complex regulates inflammatory cytokine production and secretion. As mentioned earlier, the NLRP3 inflammasome consists of a sensor (NLRP3), an adaptor (ASC), and an effector (caspase-1). Upon stimulation, NLRP3 oligomerizes and recruits ASC. Natural plant products with antioxidant and anti-inflammatory activity are expected to contribute to the regulation of inflammasome activity, and inflammasome-related studies have been reported on natural products [52–56]. Therefore, we previously investigated the use of a natural product from *Quercus* spp. As a therapeutic agent for inflammatory diseases by evaluating the production of NO and proinflammatory cytokines [18], the results of our previous study demonstrated that *Q. serrata* extract can potentially be applied for the treatment of inflammatory diseases such as arthritis treatment.

Our results in this study demonstrated that the *Q. serrata* extract evaluated here, as well as compounds 1, 4, and 5 exhibited good antioxidant ability; the major component, compound 5, was most potent in this regard. In addition, the *Q. serrata* extract and compounds 4 and 5 exerted anti-inflammatory effects. Based on these results, we evaluated whether the *Q. serrata* extract and compound 5 were able to inhibit the expression of the components of the NLRP3 inflammasome. Both the extract and compound 5 suppressed the expression of the NLRP3 inflammasome components. Western blot analysis of the NLRP3 components revealed that the suppression of the protein expression of all the three components (NLRP3, caspase-1, and ASC) by the extract and compound 5 was clearly concentration-dependent. The extract and compound 5 also showed potent antioxidant and anti-inflammatory abilities. Inhibition of collagenase activity is assessed to evaluate whether the test compound can potentially be applied for arthritis treatment. In our study, both the *Q. serrata* extract and compound 5 showed a potent collagenase inhibitory activity. There has been increased interest regarding the role of the inflammasome in arthritis. Increased secretion of NLRP3, ASC, and caspase-1 has been reported in arthritis patients, and suppression of NLRP3 activity was reported to suppress arthritis in rat models [43]. In addition, higher levels of NLRP3 and caspase-1 were observed in patients with rheumatoid arthritis than in patients with degenerative arthritis, and it was found that the administration of selective NLRP3 inhibitors reduced redness and cartilage degradation [57]. On the other hand, it has been reported that controlling the activity of NLRP3 is a good strategy for the treatment of wrist ligament injury [58]. Based on the above literature reports, it is clear that a drug that regulates the inflammasome will be beneficial in arthritis. Therefore, targeting the NLRP3 inflammasome may provide new therapeutic strategies for treating arthritis. In this study, we employed the DPPH radical scavenging activity, NO production inhibitory activity assay, and NLRP3 inflammasome component expression assay to comprehensively evaluate the bioactivity profile of the *Q. serrata* extract and its component compounds. Our results showed that the QS extract and its component compounds may have therapeutic benefits towards inflammatory diseases such as arthritis.

## 5. Conclusions

In this study, five compounds (1–5) were isolated from *Q. serrata*, namely caffeic acid (1), myricetin-3-O-cellobioside (2), phloroglucinol (3), (S)-2,3-HHDP-D-glucopyranoside (4), and pedunculagin (5). Content analysis showed that pedunculagin (5) was the main constituent of this extract. The antioxidant activity of the extract and the component compounds was evaluated by measuring DPPH radical scavenging activity. The *Q. serrata* extract and the isolated compounds (1–5) exhibited increased DPPH radical scavenging activity, and compound 5 performed the best in this assay. The anti-inflammatory activities were evaluated based on the inhibition of NO production. The *Q. serrata* extract and compounds 4 and 5 showed potential NO inhibitory activity. The *Q. serrata* extract and compound 5 potently inhibited the protein expression of the components of the NLRP3 inflammasome, including NLRP3, ASC, and caspase-1, further confirming their anti-inflammatory nature. The *Q. serrata* extract and compound 5 also showed potent collagenase inhibitory activity. The present results suggest that the *Q. serrata* extract and compound 5 are promising candidates for treating inflammatory diseases such as arthritis. However, further *in vivo* studies in arthritis animal models are required to confirm these findings.

## Figures and Tables

**Table 1:** Analysis conditions for high-performance liquid chromatography (HPLC).

*HPLC condition*			

Mobile phase	A: 0.2% acetic acid in H_2_O B: Acetonitrile (ACN)	Flow rate: 1.0 mL/min Injection vol.: 10.0 *μ*L	
Gradient profile	Time (min)	A (%)	B (%)
	0.0 10.0 35.0 55.0 59.0	95 75 50 0 0	5 25 50 100 100
*HPLC instrument*			
Controller Column Detector	Waters 2695 Separations Module Hector C18 HPLC Column (5 *μ*m, 250 × 4.6 mm) Waters 996 Photodiode Array Detector		

**Table 2:** Proportion of compounds 1–5 in the *Q. serrata* extract.

Compound	Proportion in the extract (%)
**1**	0.04
**2**	0.32
**3**	0.22
**4**	6.22
**5**	16.56

DPPH radical scavenging activity.

**Table 3:** IC_50_ value of the *Q. serrata* extract in the 2,2-diphenyl-1-picryl-hydrazyl (DPPH) radical scavenging assay.

Test sample	IC_50_ (*μ*g/mL)
*Q. serrata* extract	65.84 ± 5.79
Ascorbic acid	11.20 ± 5.97

**Table 4:** IC_50_ values of compounds 1–5 and ascorbic acid in the 2,2-diphenyl-1-picryl-hydrazyl (DPPH) radical scavenging assay.

Compound	IC_50_ (*μ*M)
Caffeic acid (1)	53.99 ± 1.91
Myricetin-3-O-cellobioside (2)	103.35 ± 2.55
Phloroglucinol (3)	71.98 ± 2.92
(S)-2,3-HHDP-D-glucopyranoside (4)	56.19 ± 4.18
Pedunculagin (5)	24.18 ± 1.06
Ascorbic acid	51.07 ± 1.47

**Table 5:** IC_50_ values of the *Q. serrata* extract and positive control L-NMMA.

Test sample	IC_50_ (*μ*g/mL)
*Q. serrata*	6.32 ± 0.19
L-NMMA	5.10 ± 0.14

L-NMMA, NG-monomethyl-L-arginine monoacetate.

**Table 6:** IC_50_ values pertaining to the inhibitory activity of compounds 1–5 on nitric oxide (NO) production.

Compound	IC_50_ (*μ*M)
**1**	158.32 ± 2.58
**2**	134.01 ± 2.63
**3**	121.22 ± 6.86
**4**	117.66 ± 2.63
**5**	111.79 ± 2.52
L-NMMA	16.85 ± 3.40

L-NMMA, NG-monomethyl-L-arginine monoacetate.

**Table 7:** Collagenase inhibitory activity of the *Q. serrata* extract.

QS Extract	Inhibitory activity (%)
25 *μ*M	99.68
12.5 *μ*M	33.39
6.25 *μ*M	15.33

**Table 8:** Collagenase inhibitory activity of compound 5.

Compound 5 (*μ*M)	Inhibitory activity (%)
25	57.52
12.5	49.07
6.25	31.49

**Table 9:** Collagenase inhibitory activity of the control.

EGCG (*μ*M)	Inhibitory activity (%)
25	85.30
12.5	72.59
6.25	59.51

## Data Availability

The data used to support the findings of this study are included within the article.

## Supplementary Materials

Supplementary Materials Supplementary Material files include the NMR spectra of five compounds isolated from *Q. serrata*. Figure S1-1: ^1^H-NMR spectrum of compound **1** (600 MHz, acetone-* d*_6_+D2O); Figure S1-2: ^13^C-NMR spectrum of compound **1** (150 MHz, acetone-* d*_6_+D_2_O); Figure S2-1: ^1^H-NMR spectrum of compound **2** (600 MHz, DMSO-*d*_6_+D_2_O); Figure S2-2: ^13^C-NMR spectrum of compound **2** (150 MHz, DMSO-*d*_6_+D_2_O); Figure S3-1: ^1^H-NMR spectrum of compound **3** (600 MHz, DMSO-*d*_6_+D_2_O); Figure S3-2: ^13^C-NMR spectrum of compound **3** (150 MHz, DMSO-*d*_6_+D_2_O); Figure S4-1: ^1^H-NMR spectrum of compound **4** (600 MHz, acetone-* d*_6_+D_2_O); Figure S4-2: ^13^C-NMR spectrum of compound **4** (150 MHz, MeOD); and Figure S5-1: ^1^H-NMR spectrum of compound **5** (600 MHz, DMSO-*d*_6_+D_2_O).
