# Supplementary figures and images for: Inhibition of the NLRP3 Inflammasome by a Quercus Serrata Extract and Isolation of the Component Compounds for the Treatment of Arthritis
Source: Evid Based Complement Alternat Med. 2022 Dec 28;2022:4428269. doi: 10.1155/2022/4428269 (PMC9812605; doi:10.1155/2022/4428269)

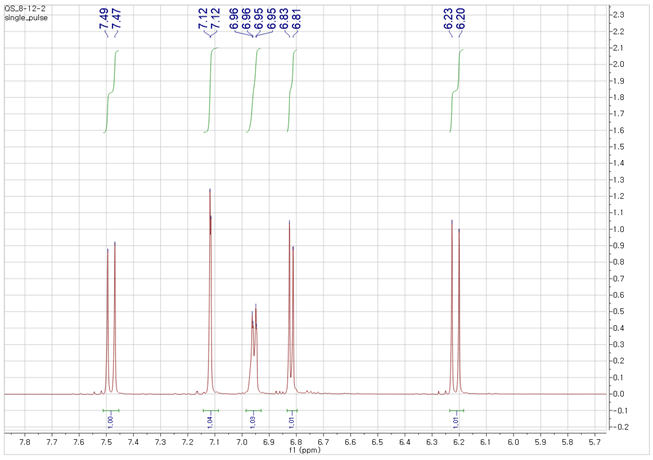

Supplement: Supplementary Materials — Supplementary Material files include the NMR spectra of five compounds isolated from Q. serrata. Figure S1-1: 1H-NMR spectrum of compound 1 (600 MHz, acetone- d6+D2O); Figure S1-2: 13C-NMR spectrum of compound 1 (150 MHz, acetone- d6+D2O); Figure S2-1: 1H-NMR spectrum of compound 2 (600 MHz, DMSO-d6+D2O); Figure S2-2: 13C-NMR spectrum of compound 2 (150 MHz, DMSO-d6+D2O); Figure S3-1: 1H-NMR spectrum of compound 3 (600 MHz, DMSO-d6+D2O); Figure S3-2: 13C-NMR spectrum of compound 3 (150 MHz, DMSO-d6+D2O); Figure S4-1: 1H-NMR spectrum of compound 4 (600 MHz, acetone- d6+D2O); Figure S4-2: 13C-NMR spectrum of compound 4 (150 MHz, MeOD); and Figure S5-1: 1H-NMR spectrum of compound 5 (600 MHz, DMSO-d6+D2O). [file 4428269.f1.zip › supplementary figures/Figure S1-1. 1H-NMR spectrum of compound 1.png]

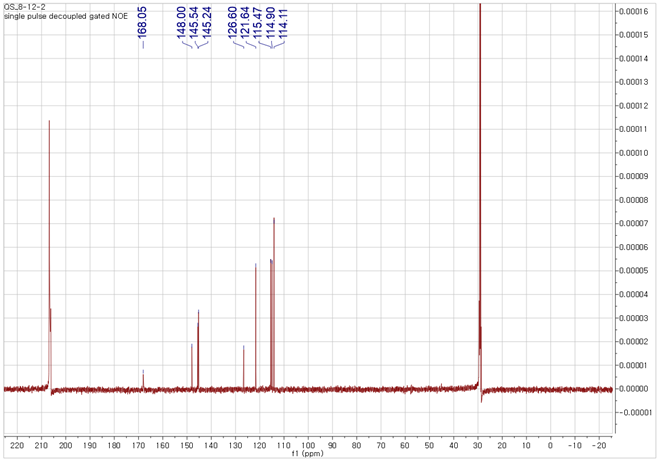

Supplement: Supplementary Materials — Supplementary Material files include the NMR spectra of five compounds isolated from Q. serrata. Figure S1-1: 1H-NMR spectrum of compound 1 (600 MHz, acetone- d6+D2O); Figure S1-2: 13C-NMR spectrum of compound 1 (150 MHz, acetone- d6+D2O); Figure S2-1: 1H-NMR spectrum of compound 2 (600 MHz, DMSO-d6+D2O); Figure S2-2: 13C-NMR spectrum of compound 2 (150 MHz, DMSO-d6+D2O); Figure S3-1: 1H-NMR spectrum of compound 3 (600 MHz, DMSO-d6+D2O); Figure S3-2: 13C-NMR spectrum of compound 3 (150 MHz, DMSO-d6+D2O); Figure S4-1: 1H-NMR spectrum of compound 4 (600 MHz, acetone- d6+D2O); Figure S4-2: 13C-NMR spectrum of compound 4 (150 MHz, MeOD); and Figure S5-1: 1H-NMR spectrum of compound 5 (600 MHz, DMSO-d6+D2O). [file 4428269.f1.zip › supplementary figures/Figure S1-2. 13C-NMR spectrum of compound 1.png]

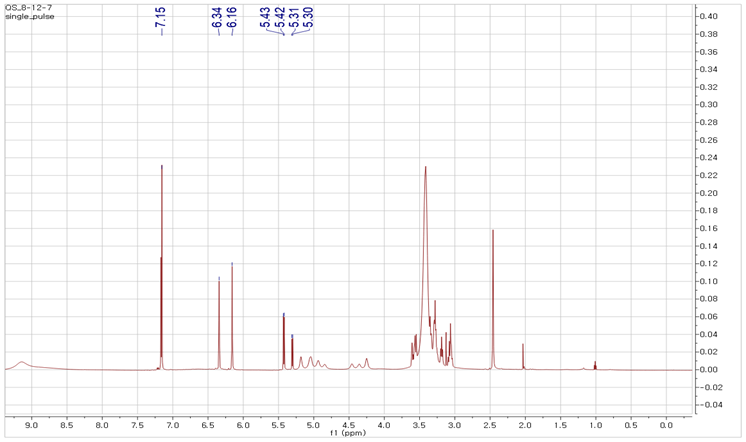

Supplement: Supplementary Materials — Supplementary Material files include the NMR spectra of five compounds isolated from Q. serrata. Figure S1-1: 1H-NMR spectrum of compound 1 (600 MHz, acetone- d6+D2O); Figure S1-2: 13C-NMR spectrum of compound 1 (150 MHz, acetone- d6+D2O); Figure S2-1: 1H-NMR spectrum of compound 2 (600 MHz, DMSO-d6+D2O); Figure S2-2: 13C-NMR spectrum of compound 2 (150 MHz, DMSO-d6+D2O); Figure S3-1: 1H-NMR spectrum of compound 3 (600 MHz, DMSO-d6+D2O); Figure S3-2: 13C-NMR spectrum of compound 3 (150 MHz, DMSO-d6+D2O); Figure S4-1: 1H-NMR spectrum of compound 4 (600 MHz, acetone- d6+D2O); Figure S4-2: 13C-NMR spectrum of compound 4 (150 MHz, MeOD); and Figure S5-1: 1H-NMR spectrum of compound 5 (600 MHz, DMSO-d6+D2O). [file 4428269.f1.zip › supplementary figures/Figure S2-1. 1H-NMR spectrum of compound 2.png]

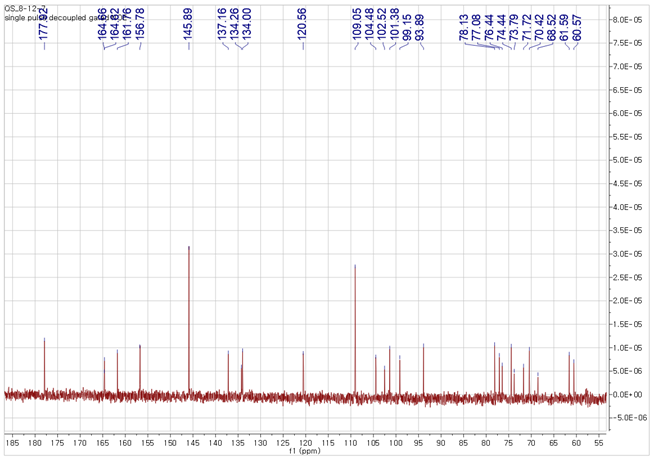

Supplement: Supplementary Materials — Supplementary Material files include the NMR spectra of five compounds isolated from Q. serrata. Figure S1-1: 1H-NMR spectrum of compound 1 (600 MHz, acetone- d6+D2O); Figure S1-2: 13C-NMR spectrum of compound 1 (150 MHz, acetone- d6+D2O); Figure S2-1: 1H-NMR spectrum of compound 2 (600 MHz, DMSO-d6+D2O); Figure S2-2: 13C-NMR spectrum of compound 2 (150 MHz, DMSO-d6+D2O); Figure S3-1: 1H-NMR spectrum of compound 3 (600 MHz, DMSO-d6+D2O); Figure S3-2: 13C-NMR spectrum of compound 3 (150 MHz, DMSO-d6+D2O); Figure S4-1: 1H-NMR spectrum of compound 4 (600 MHz, acetone- d6+D2O); Figure S4-2: 13C-NMR spectrum of compound 4 (150 MHz, MeOD); and Figure S5-1: 1H-NMR spectrum of compound 5 (600 MHz, DMSO-d6+D2O). [file 4428269.f1.zip › supplementary figures/Figure S2-2. 13C-NMR spectrum of compound 2.png]

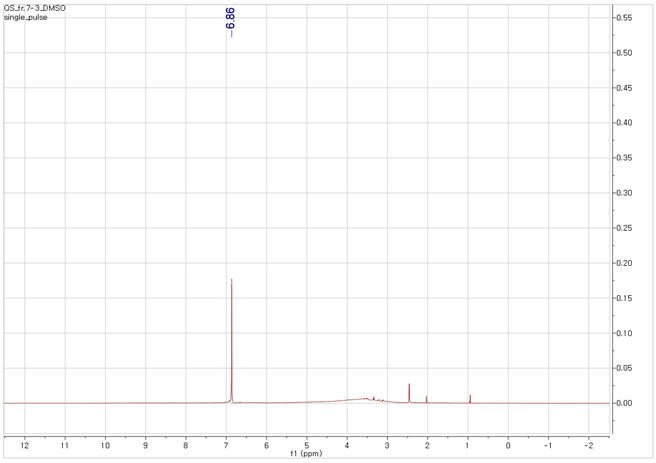

Supplement: Supplementary Materials — Supplementary Material files include the NMR spectra of five compounds isolated from Q. serrata. Figure S1-1: 1H-NMR spectrum of compound 1 (600 MHz, acetone- d6+D2O); Figure S1-2: 13C-NMR spectrum of compound 1 (150 MHz, acetone- d6+D2O); Figure S2-1: 1H-NMR spectrum of compound 2 (600 MHz, DMSO-d6+D2O); Figure S2-2: 13C-NMR spectrum of compound 2 (150 MHz, DMSO-d6+D2O); Figure S3-1: 1H-NMR spectrum of compound 3 (600 MHz, DMSO-d6+D2O); Figure S3-2: 13C-NMR spectrum of compound 3 (150 MHz, DMSO-d6+D2O); Figure S4-1: 1H-NMR spectrum of compound 4 (600 MHz, acetone- d6+D2O); Figure S4-2: 13C-NMR spectrum of compound 4 (150 MHz, MeOD); and Figure S5-1: 1H-NMR spectrum of compound 5 (600 MHz, DMSO-d6+D2O). [file 4428269.f1.zip › supplementary figures/Figure S3-1. 1H-NMR spectrum of compound 3.png]

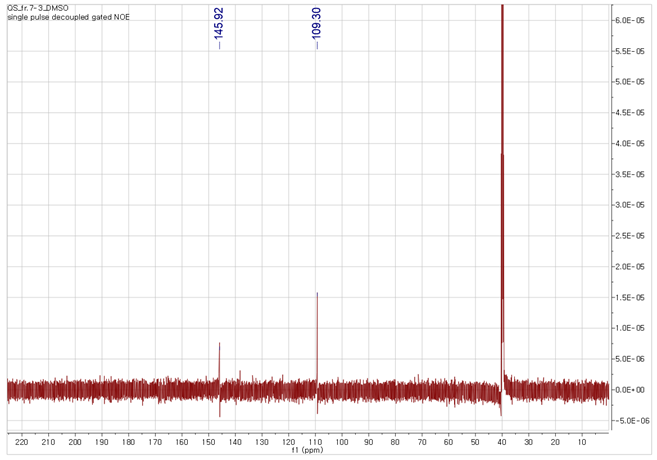

Supplement: Supplementary Materials — Supplementary Material files include the NMR spectra of five compounds isolated from Q. serrata. Figure S1-1: 1H-NMR spectrum of compound 1 (600 MHz, acetone- d6+D2O); Figure S1-2: 13C-NMR spectrum of compound 1 (150 MHz, acetone- d6+D2O); Figure S2-1: 1H-NMR spectrum of compound 2 (600 MHz, DMSO-d6+D2O); Figure S2-2: 13C-NMR spectrum of compound 2 (150 MHz, DMSO-d6+D2O); Figure S3-1: 1H-NMR spectrum of compound 3 (600 MHz, DMSO-d6+D2O); Figure S3-2: 13C-NMR spectrum of compound 3 (150 MHz, DMSO-d6+D2O); Figure S4-1: 1H-NMR spectrum of compound 4 (600 MHz, acetone- d6+D2O); Figure S4-2: 13C-NMR spectrum of compound 4 (150 MHz, MeOD); and Figure S5-1: 1H-NMR spectrum of compound 5 (600 MHz, DMSO-d6+D2O). [file 4428269.f1.zip › supplementary figures/Figure S3-2. 13C-NMR spectrum of compound 3.png]

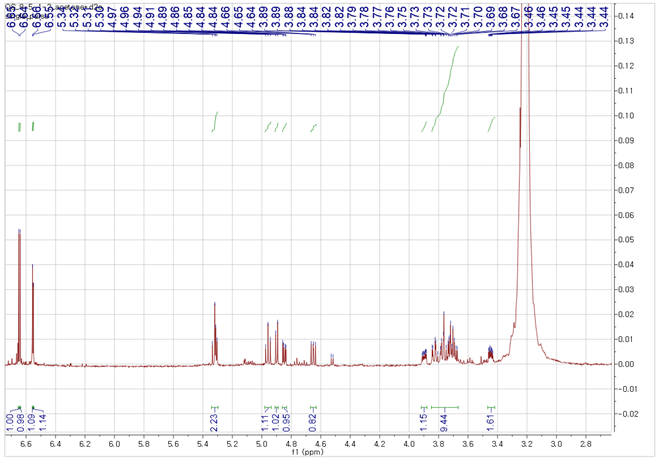

Supplement: Supplementary Materials — Supplementary Material files include the NMR spectra of five compounds isolated from Q. serrata. Figure S1-1: 1H-NMR spectrum of compound 1 (600 MHz, acetone- d6+D2O); Figure S1-2: 13C-NMR spectrum of compound 1 (150 MHz, acetone- d6+D2O); Figure S2-1: 1H-NMR spectrum of compound 2 (600 MHz, DMSO-d6+D2O); Figure S2-2: 13C-NMR spectrum of compound 2 (150 MHz, DMSO-d6+D2O); Figure S3-1: 1H-NMR spectrum of compound 3 (600 MHz, DMSO-d6+D2O); Figure S3-2: 13C-NMR spectrum of compound 3 (150 MHz, DMSO-d6+D2O); Figure S4-1: 1H-NMR spectrum of compound 4 (600 MHz, acetone- d6+D2O); Figure S4-2: 13C-NMR spectrum of compound 4 (150 MHz, MeOD); and Figure S5-1: 1H-NMR spectrum of compound 5 (600 MHz, DMSO-d6+D2O). [file 4428269.f1.zip › supplementary figures/Figure S4-1. 1H-NMR spectrum of compound 4.png]

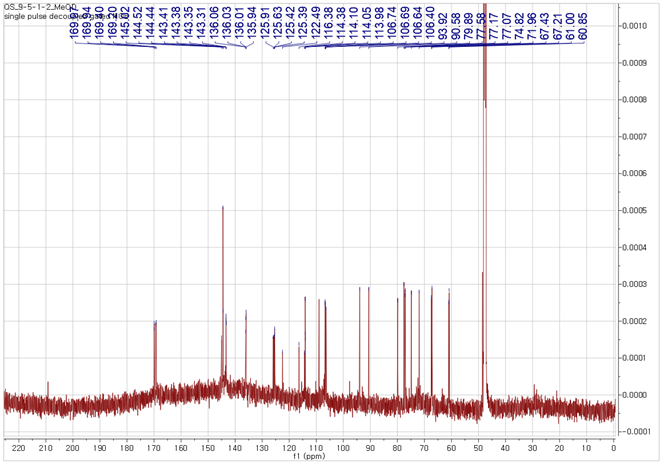

Supplement: Supplementary Materials — Supplementary Material files include the NMR spectra of five compounds isolated from Q. serrata. Figure S1-1: 1H-NMR spectrum of compound 1 (600 MHz, acetone- d6+D2O); Figure S1-2: 13C-NMR spectrum of compound 1 (150 MHz, acetone- d6+D2O); Figure S2-1: 1H-NMR spectrum of compound 2 (600 MHz, DMSO-d6+D2O); Figure S2-2: 13C-NMR spectrum of compound 2 (150 MHz, DMSO-d6+D2O); Figure S3-1: 1H-NMR spectrum of compound 3 (600 MHz, DMSO-d6+D2O); Figure S3-2: 13C-NMR spectrum of compound 3 (150 MHz, DMSO-d6+D2O); Figure S4-1: 1H-NMR spectrum of compound 4 (600 MHz, acetone- d6+D2O); Figure S4-2: 13C-NMR spectrum of compound 4 (150 MHz, MeOD); and Figure S5-1: 1H-NMR spectrum of compound 5 (600 MHz, DMSO-d6+D2O). [file 4428269.f1.zip › supplementary figures/Figure S4-2. 13C-NMR spectrum of compound 4.png]

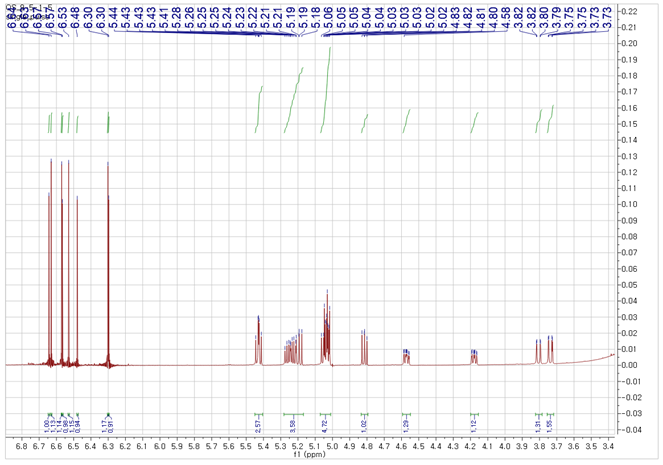

Supplement: Supplementary Materials — Supplementary Material files include the NMR spectra of five compounds isolated from Q. serrata. Figure S1-1: 1H-NMR spectrum of compound 1 (600 MHz, acetone- d6+D2O); Figure S1-2: 13C-NMR spectrum of compound 1 (150 MHz, acetone- d6+D2O); Figure S2-1: 1H-NMR spectrum of compound 2 (600 MHz, DMSO-d6+D2O); Figure S2-2: 13C-NMR spectrum of compound 2 (150 MHz, DMSO-d6+D2O); Figure S3-1: 1H-NMR spectrum of compound 3 (600 MHz, DMSO-d6+D2O); Figure S3-2: 13C-NMR spectrum of compound 3 (150 MHz, DMSO-d6+D2O); Figure S4-1: 1H-NMR spectrum of compound 4 (600 MHz, acetone- d6+D2O); Figure S4-2: 13C-NMR spectrum of compound 4 (150 MHz, MeOD); and Figure S5-1: 1H-NMR spectrum of compound 5 (600 MHz, DMSO-d6+D2O). [file 4428269.f1.zip › supplementary figures/Figure S5-1. 1H-NMR spectrum of compound 5.png]
